# Diagnostic utility of ultrasound in pediatric nasal bone fractures: a systematic review and meta-analysis

**DOI:** 10.1007/s10140-024-02225-1

**Published:** 2024-03-28

**Authors:** Amir Hassankhani, Melika Amoukhteh, Payam Jannatdoust, Parya Valizadeh, Delaram J. Ghadimi, Nikoo Saeedi, Mobina Fathi, Shirin Yaghoobpoor, Paniz Adli, Pauravi S. Vasavada, Ali Gholamrezanezhad

**Affiliations:** 1 Department of Radiology, Keck School of Medicine, University of Southern California (USC), 1441 Eastlake Ave Ste 2315, Los Angeles, CA 90089 USA; 2 Department of Radiology, Mayo Clinic, Rochester, MN USA; 3 School of Medicine, Tehran University of Medical Sciences, Tehran, Iran; 4 School of Medicine, Shahid Beheshti University of Medical Sciences, Tehran, Iran; 5 Student Research Committee, School of Medicine, Islamic Azad University, Mashhad Branch, Mashhad, Iran; 6 Student Research Committee, School of Medicine, Shahid Beheshti University of Medical Sciences, Tehran, Iran; 7 College of Letters and Science, University of California, Berkeley, CA USA; 8 Department of Radiology, University Hospitals Case Medical Center, Case Western Reserve University School of Medicine, Cleveland, OH USA

**Keywords:** Pediatrics, Ultrasonography, Nasal bone, Fractures

## Abstract

Ultrasonography, a radiation-free and cost-effective modality, stands out as a promising tool for evaluating nasal bone fractures. Despite limited literature on its pediatric application, there is an increasing recognition of its potential to enhance diagnostic precision. To evaluate the diagnostic efficacy of ultrasound in detecting pediatric nasal bone fractures. Employing established guidelines, a systematic review and meta-analysis were conducted through a comprehensive literature search in PubMed, Scopus, Web of Science, and Embase databases until December 5, 2023. Inclusion criteria encompassed studies reporting diagnostic accuracy measures of ultrasound in pediatric patients with nasal bone fractures. Data extraction and analysis were undertaken for the selected studies. Involving four studies with 277 patients, ultrasound demonstrated a pooled sensitivity of 66.1% (95% CI: 35.1-87.5%) and specificity of 86.8% (95% CI: 80.1-91.4%) in diagnosing pediatric nasal fractures. The area under the receiver operating characteristic curve (AUC) was 0.88 (95% CI: 0.72–0.93). After excluding an outlier study, sensitivity and specificity increased to 78.0% (95% CI: 65.6-86.9%) and 87.8 (95% CI: 78.1-93.6%), respectively, with an AUC of 0.79 (95% CI: 0.75–0.94). Pooled positive and negative likelihood ratios were 5.11 (95% CI: 2.12–9.15) and 0.40 (95% CI: 0.14–0.77) before exclusion and 6.75 (95% CI: 3.47–12.30) and 0.26 (95% CI: 0.15–0.40) after exclusion of an outlier study, respectively. This study highlighted ultrasonography’s utility in diagnosing pediatric nasal bone fractures with high accuracy and specificity. However, caution is advised in relying solely on ultrasound due to suboptimal overall diagnostic performance, evident in likelihood ratios.

**Supplementary Information:**

The online version contains supplementary material available at 10.1007/s10140-024-02225-1.

## Introduction

Pediatric nasal bone fractures are highly prevalent, constituting 40–50% of facial fractures in children [1–3]. The untreated consequences of these fractures can lead to both cosmetic and functional impairments, underscoring the critical need for accurate and timely diagnosis [4, 5].

While physical examination is widely acknowledged as the gold standard for diagnosing nasal fractures [1, 6], challenges arise when significant post-traumatic swelling occurs, hindering the prompt identification of deformities [7], especially in younger age patients who have higher amount of cartilage in nasal structures [4], [5]. Furthermore, the subjective nature of physical examination necessitates the exploration of supplementary objective imaging techniques, particularly for comprehensive documentation, often for legal purposes. This requirement is accentuated in pediatric cases, where the inherent challenges of patient non-compliance further complicate the efficacy of physical examinations [4–6]. Traditional imaging methods, including X-rays and computed tomography (CT) scans, present limitations in sensitivity, specificity, and concerns about radiation exposure [8, 9]. In this context, ultrasonography emerges as a promising method for evaluating pediatric nasal bone fractures. This imaging modality can detect skeletal deformation along three axes without radiation exposure. Its cost-effectiveness, especially in resource-limited settings, and the improved quality of results due to the absence of an air gap between the transducer and bones make it a valuable diagnostic tool. Furthermore, owing to the portability of ultrasound devices, their utility extends beyond hospital environments, proving invaluable in situations involving multiple trauma patients. This advantage enables swift assessment and diagnosis without the constraints associated with bulkier imaging modalities [1, 10, 11].

Prior research has investigated the effectiveness of ultrasound in the diagnosis of nasal bone fractures, yielding promising outcomes [11–13]. However, the literature on its application in detecting nasal bone fractures in the pediatric population is limited. The present systematic review and meta-analysis aim to evaluate the diagnostic utility of ultrasound for nasal bone fractures, examining its sensitivity, specificity, and other diagnostic accuracy measures in the pediatric population.

## Methods

This systematic review adheres to the guidelines established in the Preferred Reporting Items for Systematic Reviews and Meta-Analyses (PRISMA) statement [14]. The literature search, initiated on December 5, 2023, spanned four major databases: PubMed, Scopus, Web of Science, and Embase. Distinct search terms tailored for each database included (“nasal” OR “midfacial”) AND (“fracture*”) AND (“sonograph*” OR “ultrasonograph*” OR “ultrasound” OR “POCUS”) AND (“pediatric*” OR “paediatric*” OR “child*” OR “neonat*” OR “infant*” OR “toddler*” OR “preschool” OR “pre-school” OR “juvenile” OR “young adult*”). Additionally, a meticulous manual examination of references within the selected studies ensured comprehensive coverage. The review process involved a thorough evaluation of each article’s title, abstract, and/or full text, conducted independently by two co-authors. Uncertainties or ambiguities were addressed through consultation with a senior co-author. Deduplication, screening, and data extraction were facilitated by the AutoLit platform, developed by Nested Knowledge in St. Paul, Minnesota, USA.

All studies relevant to the diagnostic accuracy of ultrasound in pediatric patients (under 21 years old) were considered for inclusion if they presented at least one of the following diagnostic measures: sensitivity, specificity, positive predictive value (PPV), negative predictive value (NPV), likelihood ratio (LR), diagnostic odds ratio (DOR), and area under the receiver operating characteristic curve (AUC). No restrictions were imposed on publication date, country of origin, patient characteristics, reference standard type, or study design. Non-English literature, case reports, case series with fewer than five eligible patients, conference abstracts, editorial comments, and review articles were excluded from the study.

Details extracted from each qualifying paper included the first author’s name, publication year, study design, sample size, participant and fracture characteristics, reference standard modality, ultrasound operator, type of ultrasound device, type of ultrasound probe, image acquisition methods, ultrasound features indicating nasal bone fractures, and diagnostic accuracy measures of ultrasound.

The quality assessment utilized the Diagnostic Accuracy Studies-2 (QUADAS-2) tool to evaluate included studies’ quality [15]. Independent assessments for potential bias and concerns regarding applicability were conducted for the four primary domains of the QUADAS-2 tool: patient selection, index test, reference standard, and flow and timing. Specific criteria outlined in the tool, such as the representativeness of the study population, blinding of test results, and completeness of outcome data, informed evaluations for each domain. Ratings of “low,” “high,” or “unclear” were assigned to determine the overall rigor and reliability of the evidence synthesis.

### Statistical analysis

The analysis employed a random effects diagnostic test accuracy (DTA) model, specifically utilizing the bivariate model proposed by Reitsma et al. [16]. Summary Receiver Operating Characteristic (SROC) curves were generated based on the bivariate meta-analysis data, with study-specific estimates weighted in the random effects univariate DOR model. The AUC and its confidence interval (CI) for each subgroup were determined through 2000 sample bootstrapping, utilizing the bivariate model [17].

Heterogeneity assessment relied on the I^2^ metric, following the method outlined by Holling et al. [18], where an I^2^ CI exceeding 50% signified significant heterogeneity. Sensitivity analyses using the DOR univariate meta-analysis were conducted to identify potential outliers in the presence of significant heterogeneity. If outliers were identified, a re-analysis was performed to validate the results. The clinical relevance of the findings was explored using Fagan plots and likelihood ratio scattergrams, where positive likelihood ratios above 10 indicated confirmation suitability, and negative ratios below 0.1 suggested suitability for exclusion. Fagan nomograms were constructed for assumed pre-test prevalences of 25%, 50%, and 75%, based on the bivariate Reitsma model, as detailed by Zwinderman et al. [19].

All analyses were executed in R (version 4.3.2, R Foundation for Statistical Computing, Vienna, Austria), utilizing packages such as “mada,” “dmetatools” [20], “Metafor” [21], and “meta” [22].

## Results

### Screening and selection of articles

**Fig. 1:**
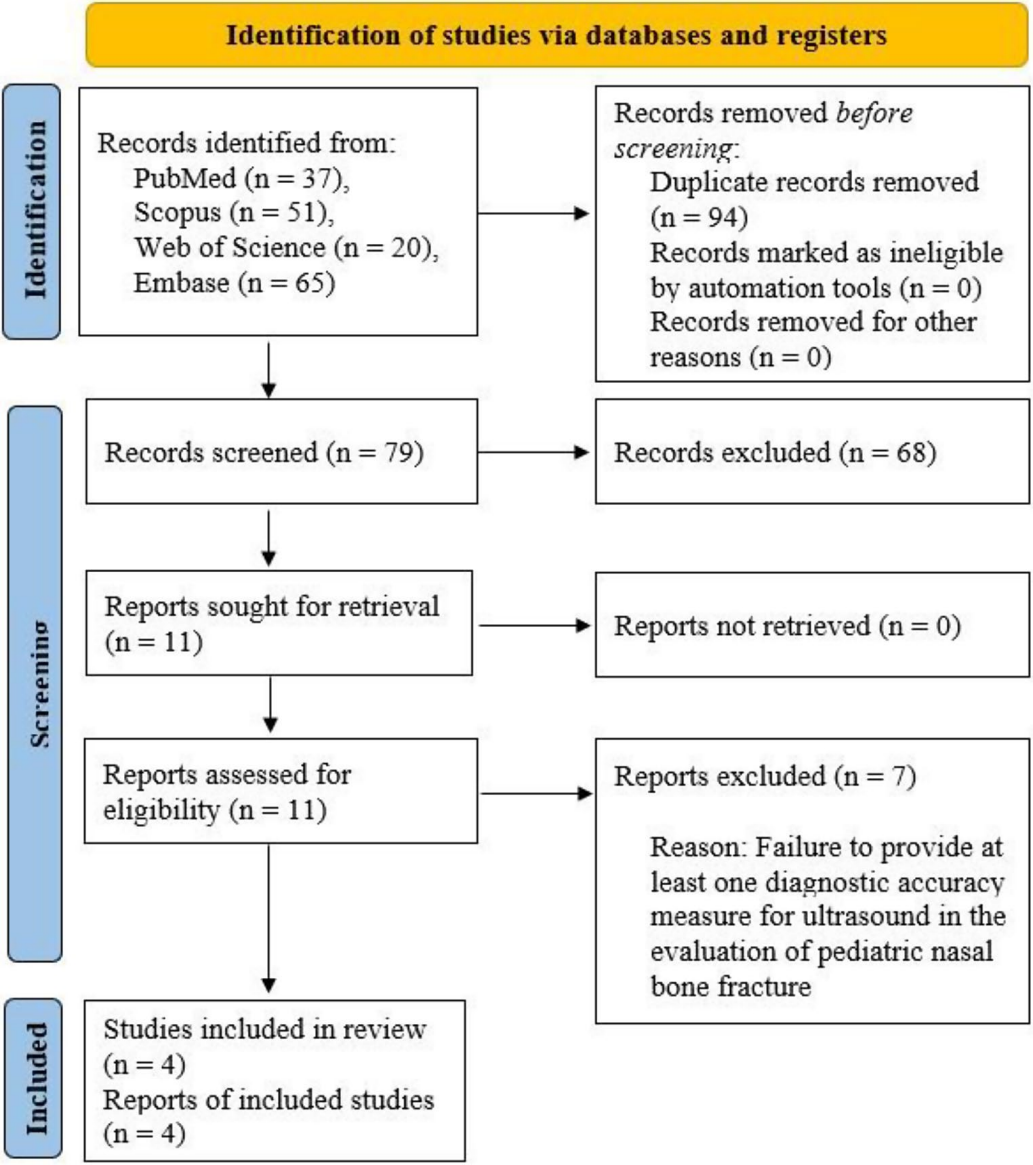
PRISMA flow diagram showing the review process. PRISMA: Preferred Reporting Items for Systematic Reviews and Meta-Analyses

The initial phase involved a systematic literature search using a predefined strategy, resulting in the identification of 173 articles. After the removal of duplicates, 79 papers underwent screening based on title and abstract. Subsequently, 68 articles were excluded during this initial screening phase. The full text of the remaining 11 papers underwent review. Following a thorough examination, 7 articles were excluded due to insufficient numerical data, preventing the calculation of required values for conducting diagnostic test accuracy meta-analysis. Eventually, 4 articles that met the inclusion criteria were identified and included in the study. The screening process and eligibility criteria were in accordance with PRISMA guidelines, and a visual representation is provided in Fig. 1.

### Study and patient characteristics

The meta-analysis incorporated four studies that evaluated the efficacy of ultrasound in diagnosing pediatric nasal bone fractures. Together, these studies investigated 277 pediatric patients, employing various ultrasound devices. The inclusion criteria across studies typically involved participants aged ≤ 18 with isolated nasal trauma, excluding cases with multiple traumas. The reference tests in all included studies relied on physical examination; however, the testing protocols varied between examinations conducted by emergency physicians [8] and those carried out by otolaryngologists [1, 6] and plastic surgeons [9]. Additional information regarding the studies, characteristics of the patients, and details of the ultrasound examinations can be found in Table 1.

**Table 1:** Characteristics of the included studies

Author, Year	Country	Study design	Age, years	Patients N (F%)	Inclusion criteria	Imaging method/criteria	US device	Probe, frequency	Performer	Setting	Reference test	Sensitivity (%)	Specificity (%)
Dogan, [8]	Turkey	Prospective cohort	7.44 ± 5.05 (Mean ± SD)	133 (34.6)	Participants aged ≤ 18 with isolated nasal trauma, GCS of 15, presenting at the ED without multiple traumas or an indication for CT scan	Bedside US examinations in transverse, longitudinal, and oblique directions	Toshiba Xario XG	Linear, 7.2–14 MHz	Radiologist	Hospital/ED	EP’s physical examination	22.5	83.1
Gökçen, [6]	Turkey	Cross-sectional prospective	8 (5?13) (Median (IQR))	31 (25.8)	Participants aged ≤ 18 with nasal trauma, GCS of 15, without a prior history of nasal fracture	US examinations on patients in the supine position, using the transverse, oblique and longitudinal planes	HM70A with Plus ultrasound system (Samsung Medison Co., Ltd, Seoul, Korea)	Linear, 7-12 MHz	Trained EP	Hospital/ED	Otolaryngologist’s examination	72.2	76.9
Tamada, [9]	Japan	Prospective cohort	6.66 (Mean)	63 (31.7)	Individuals under 18 presenting to the ED with facial trauma and clinical indications of nasal bone fracture, with no other facial bone fractures or previous nasal fractures recorded	US examinations were conducted on patients in a supine position, utilizing transverse, oblique, and longitudinal planes. The diagnosis of a nasal fracture on US scans was made by criteria such as irregularities in the bone structure, asymmetry, or depression of the nasal bone	SonoSite M-Turbo manufactured by FUJIFILM SonoSite Inc, Tokyo, Japan	N/A	EP	Hospital/ED and outpatient clinic	Clinical examination in department of plastic and reconstructive surgery + CT scan	75	92.3
Noy, [1]	Israel	Prospective cohort	11 (6-15) (Median (IQR))	50 (28)	Participants aged ≤ 18 with isolated nasal trauma, presenting at the ED, without prior history of nasal trauma, open wound, without other head and neck trauma or an indication for CT scan	US examinations were conducted on patients in a supine position, utilizing transverse, oblique, and longitudinal planes. The diagnosis of a nasal fracture on US scans was made by criteria such as an interruption in the continuity of the nasal bone and visual evidence of bone displacement.	HM70A with Plus ultrasound system (Samsung Medison Co., Ltd, Seoul, Korea)	Linear, 7–12 MHz	Radiologist & EP	Hospital/ED	Otolaryngologist’s examination	90	89

CT: computed tomography, ED: emergency department, EP: emergency physician, F: female, GCS: Glasgow coma scale, IQR: interquartile range, N/A: no answer, N: number, SD: standard deviation, US: ultrasound

### Quality assessment

The quality assessment of the included studies is detailed in Table 2. These studies primarily exhibited bias related to the reference standard test, which stemmed from the use of physical examination, an inherently subjective method reliant on the examiner’s expertise. Furthermore, two studies [6, 8] in the review did not clearly specify sonographic criteria for diagnosing nasal fractures. Additionally, one study [9] exclusively employed CT scans as a reference test for patients with suspected nasal fractures in ultrasound results, rather than uniformly applying it to all patients. In summary, the review suggests a moderate overall quality of the included studies.

**Table 2:** Quality assessment of the included studies

Author	Q1	Q2	Q3	Q4	Q5	Q6	Q7	Q8	Q9	Q10	Q11	Q12	Q13	Q14	Q15	Q16	Q17
Dogan, [8]	Yes	Yes	Yes	No	No	Unclear	Unclear	No	No	Unclear	Yes	Yes	No	Unclear	Yes	Yes	No
Gökçen, [6]	Yes	Yes	Yes	No	No	Unclear	Unclear	No	No	Yes	Yes	Yes	No	Yes	Yes	Yes	No
Tamada, [9]	Yes	Yes	Yes	No	No	No	Yes	Unclear	No	Yes	No	Yes	No	Yes	No	Yes	Yes
Noy, [1]	Yes	Yes	Yes	No	No	Yes	Yes	No	No	Yes	Yes	Yes	No	Yes	Yes	Yes	No

Q1. Was a consecutive or random sample of patients enrolled?

Q2. Was a case-control design avoided?

Q3. Did the study avoid inappropriate exclusions?

Q4. Could the selection of patients have introduced bias?

Q5. Are there concerns that the included patients and setting do not match the review question?

Q6. Were the index test results interpreted without knowledge of the results of the reference standard?

Q7. If a threshold was used, was it pre-specified?

Q8. Could the conduct or interpretation of the index test have introduced bias?

Q9. Are there concerns that the index test, its conduct, or interpretation differ from the review question?

Q10. Are the reference standards likely to correctly classify the target condition?

Q11. Were the reference standard results interpreted without knowledge of the results of the index tests?

Q12. Could the reference standard, its conduct, or its interpretation have introduced bias?

Q13. Are there concerns that the target condition as defined by the reference standard does not match the question?

Q14. Was there an appropriate interval between index test and reference standard?

Q15. Did all patients receive the same reference standard?

Q16. Were all patients included in the analysis?

Q17. Could the patient flow have introduced bias?

### Meta-analysis

**Fig. 2:**
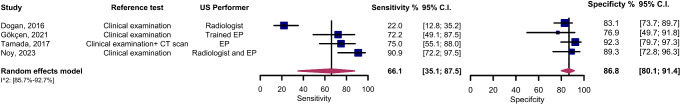
Forest plot and summary statistics of the diagnostic test accuracy (DTA) meta-analysis incorporating all included studies. CI. Confidence interval, EP. Emergency practitioner

**Fig. 3:**
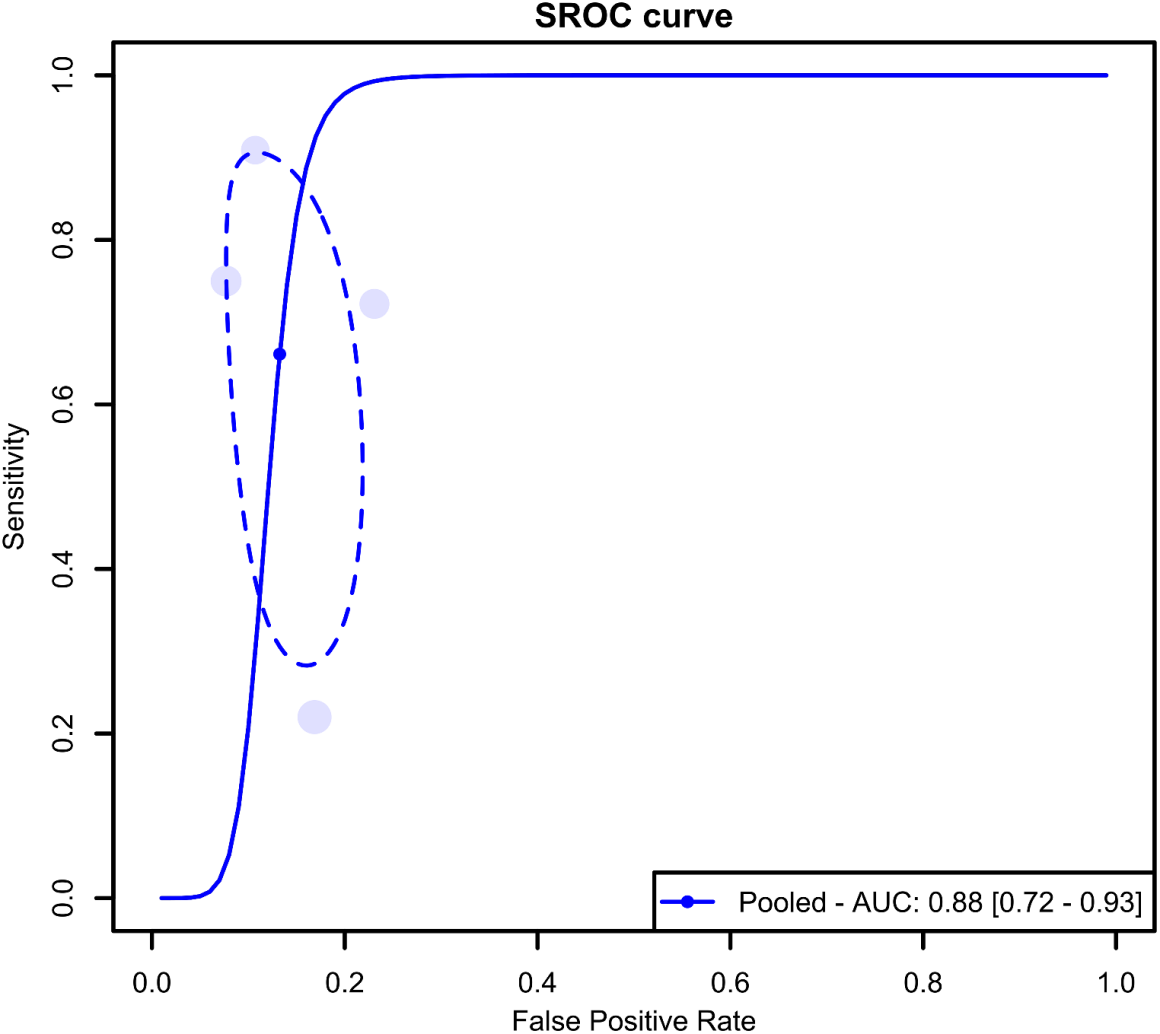
Summary receiver operating characteristic curve (SROC) for the diagnostic test accuracy (DTA) meta-analysis encompassing all included studies. AUC. Area under the curve. SROC. Summary receiver operating characteristic

The meta-analysis results from four studies assessing the accuracy of ultrasonography for pediatric nasal bone fractures revealed pooled sensitivity and specificity values of 66.1% (95% CI: 35.1-87.5%) and 86.8% (95% CI: 80.1-91.4%), as illustrated in Fig. 2. The AUC for the SROC curve was 0.88 (95% CI: 0.72–0.93), as depicted in Fig. 3.

**Fig. 4:**
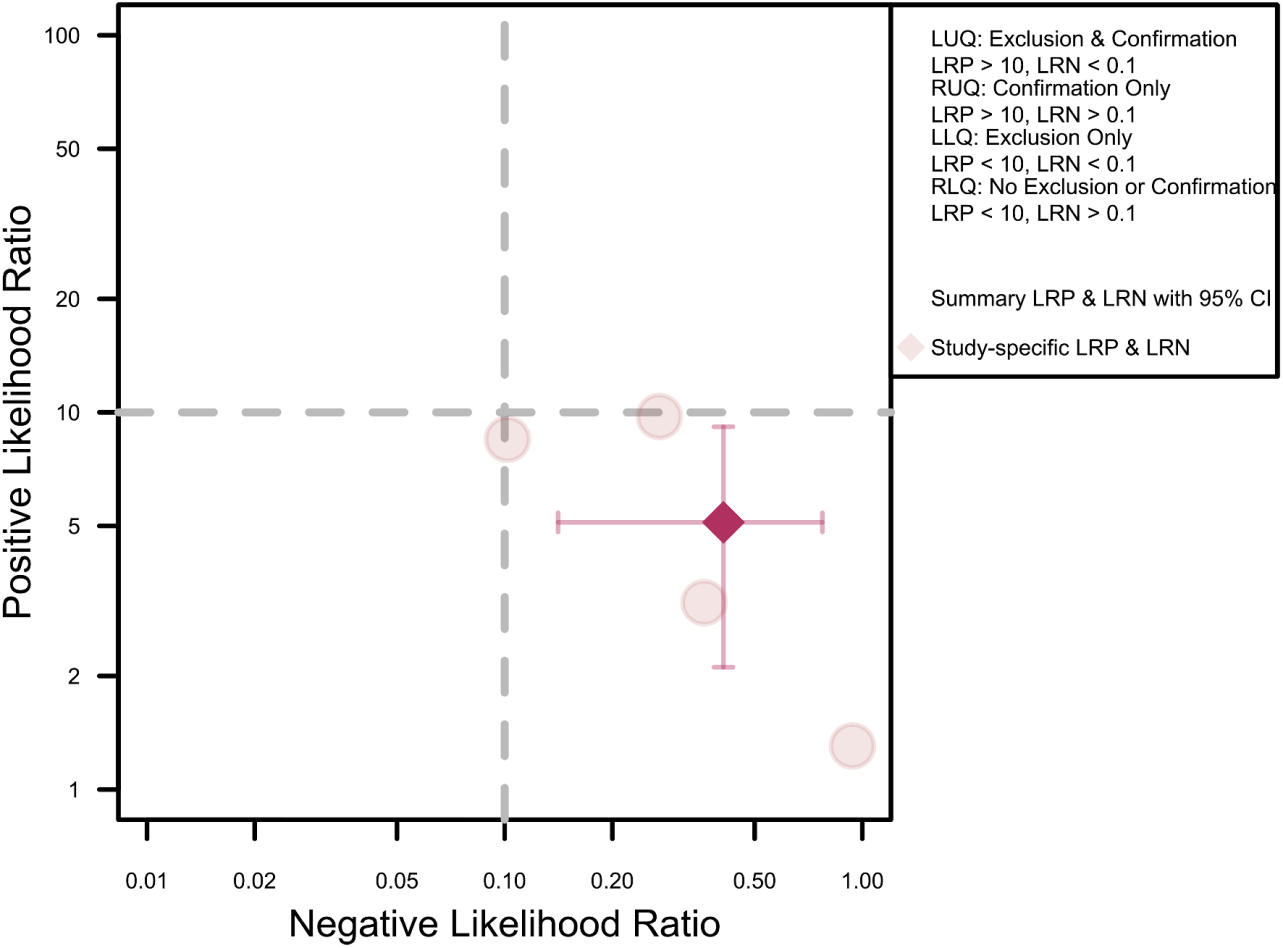
Likelihood ratio scattergram of included studies indicating low to moderate test performance suboptimal for both exclusion and confirmation purposes. LLQ. Left lower quadrant. LRN. Likelihood ratio, negative. LRP. Likelihood ratio, positive. LUQ. Left upper quadrant. RLQ. Right lower quadrant. RUQ. Right upper quadrant

Figure 4 presents a scattergram of positive and negative likelihood ratios, indicating a test performance ranging from low to moderate. This performance level is suboptimal for both exclusion and confirmation purposes. The pooled positive and negative likelihood ratios were 5.11 (95% CI: 2.12–9.15) and 0.40 (95% CI: 0.14–0.77), respectively.

**Fig. 5:**
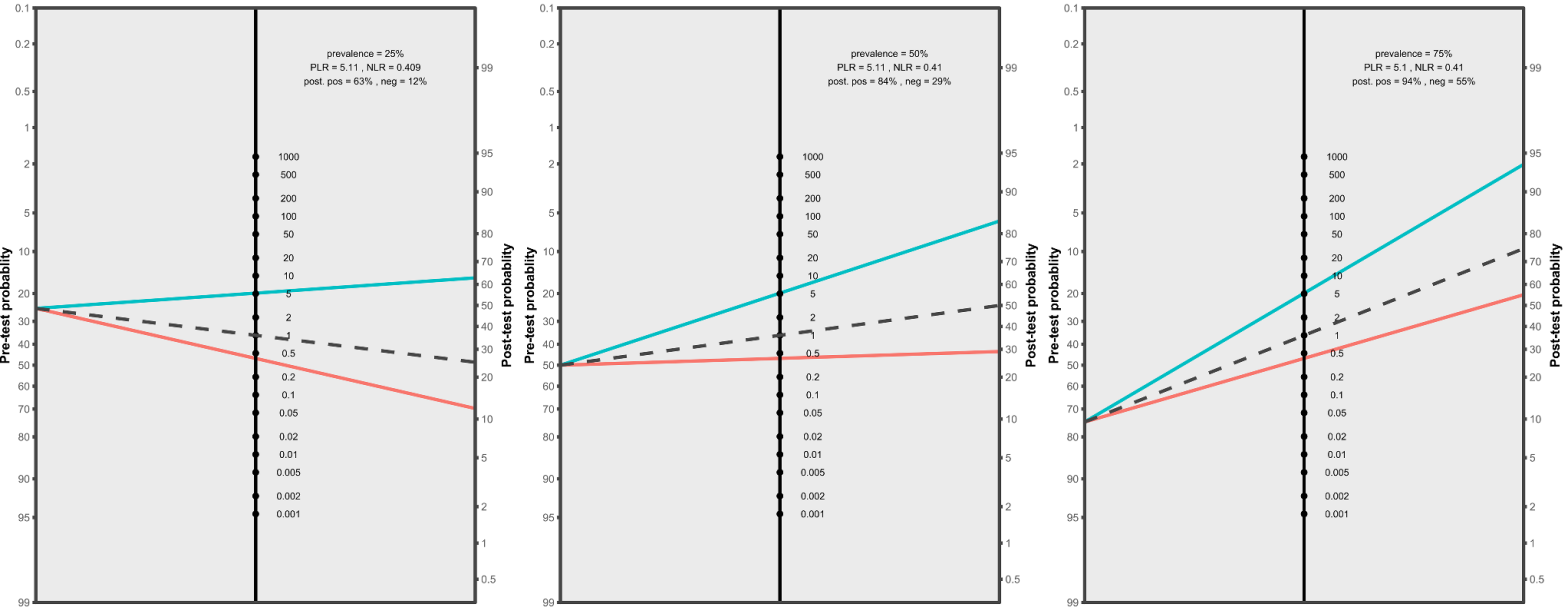
Fagan plot analysis utilizing summary positive and negative likelihood ratio results from the meta-analysis of all included studies, considering hypothetical pre-test probabilities of 25%, 50%, and 75%. PLR. Positive likelihood ratio. NLR. Negative likelihood ratio. Neg. Negative. Pos. Positive

In the Fagan plot study, considering pre-test probabilities of 25%, 50%, and 75% for pediatric nasal bone fractures, the positive post-test probabilities are 63%, 84%, and 94%, while the negative post-test probabilities are 12%, 29%, and 55%, respectively (Fig. 5).

The meta-analysis revealed substantial heterogeneity, as illustrated in Fig. 2. To pinpoint potential outliers and explore the origin of this heterogeneity, an influential analysis was undertaken. This analysis identified the study by Dogan et al. [8] as a significant outlier (Supplementary Fig. 1).

### Meta-analysis following the exclusion of an outlier study

The meta-analysis of the three remaining studies revealed pooled sensitivity and specificity values of 78.0% (95% CI: 65.6-86.9%) and 87.8 (95% CI: 78.1-93.6%), with a moderate level of heterogeneity, as depicted in Supplementary Fig. 2. The AUC for the SROC curve was 0.79 (95% CI: 0.75–0.94), as shown in Supplementary Fig. 3.

Supplementary Fig. 4 presents a scattergram of positive and negative likelihood ratios after excluding one outlier study, indicating a test performance ranging from low to moderate, which is suboptimal for both exclusion and confirmation purposes. The pooled positive and negative likelihood ratios were 6.75 (95% CI: 3.47–12.30) and 0.26 (95% CI: 0.15–0.40), respectively.

In the Fagan plot study, considering pre-test probabilities of 25%, 50%, and 75% for pediatric nasal bone fractures, the positive post-test probabilities are 69%, 87%, and 95%, while the negative post-test probabilities are 8%, 20%, and 44%, respectively (Supplementary Fig. 5).

## Discussion

In this systematic review and meta-analysis, ultrasonography exhibited a significant level of diagnostic accuracy in identifying nasal bone fractures in children. Importantly, even with the inclusion of more homogeneous studies, this finding remained consistent, as evidenced by a minimal difference of approximately 0.10 in the AUC. The analysis revealed that the pooled specificity of ultrasonography was significantly higher than its pooled sensitivity, both across the four included studies and even after excluding one outlier study. However, the test overall performance, as indicated by likelihood ratios, was observed to be low to moderate for both exclusion and confirmation purposes.

A meta-analysis involving 1480 patients, with no age restrictions, revealed that ultrasonography achieved a pooled sensitivity of 87.2% and specificity of 87.4% in diagnosing nasal bone fractures. The study suggests that ultrasonography surpasses plain radiography in sensitivity, specificity, PPV, and NPV. Moreover, CT scans showed only marginally higher performance indices compared to ultrasonography, except for specificity. This implies that, overall, the diagnostic accuracy for nasal bone fractures appears comparable between these studied imaging techniques [12]. In a separate meta-analysis centered on facial bone fractures, utilizing CT scans as the reference standard and with no age restrictions, ultrasonography exhibited remarkable sensitivity and specificity rates of 99% and 94%, respectively, in diagnosing nasal bone fractures. These results emphasize the strong diagnostic capabilities of ultrasonography for nasal bone fractures compared to CT scans [13].

Our findings indicated a lower pooled sensitivity compared to the two meta-analyses mentioned earlier. It is important to acknowledge that ultrasonography encounters inherent limitations in assessing bones during childhood. Nasal bones in younger children are less prominent and not fully ossified. The ongoing process of ossification results in anatomical variations during different stages of childhood, potentially contributing to the increased difficulty of utilizing ultrasonography for fracture detection [23]. Additionally, a study involving 423 patients with nasal bone fractures revealed significant differences in the most prevalent fracture types between children under 12 years old and adults. In this study, Yabe et al. observed that the most common type of nasal bone fracture in children was unilateral bone displacement without posterior shift, whereas bilateral displacement was more prevalent in older patients [24]. Moreover, the diagnosis of nasal bone fractures in children presents greater challenges due to factors such as smaller body size and reduced cooperativeness compared to adults [25].

In the current study, the pooled positive and negative likelihood ratios suggest that ultrasound’s overall diagnostic performance is approximately moderate but falls short of an ideal level. These findings emphasize the importance of exercising caution when solely relying on ultrasound for definitive diagnostic decisions in pediatric nasal bone fractures. There is potential value in supplementing ultrasound with other imaging modalities or diagnostic approaches. This also emphasizes the need for additional research and refinement to improve diagnostic accuracy and reliability of ultrasound in this context.

In our analysis, the study conducted by Dogan et al. emerged as an outlier, leading to an 11.8% reduction in the pooled sensitivity in our findings. Their study reported a sensitivity of 22.5% and a specificity of 83.1%. They attributed the low sensitivity to the younger age and higher number of their patients. Additionally, the determination of the presence of a fracture relied on a physical examination by an emergency physician specialist, while the sonography was performed by a radiologist. The clarity of whether the radiologists were aware of the diagnosis during the sonography was not provided [8]. Furthermore, their study exclusively included patients with isolated nasal fractures who did not meet the criteria for a CT scan. It is worth mentioning that in two other included studies, the diagnosis was established through the physical examination by an otolaryngologist [1, 6]. Tamada et al., on the other hand, considered serial physical examinations by emergency physicians or general pediatric registrars as the ground truth. Additionally, they followed patients with a negative physical examination for nasal fracture a few days after the initial visit. If symptoms persisted, a second ultrasonography was conducted, confirming a nasal fracture in four more patients and raising the sensitivity to 91.7% [9]. However, we included only the initial results to align with the approach taken in other included studies.

It is also important to emphasize that enhancing the diagnostic performance of ultrasonography can be achieved by incorporating physical signs of a nasal bone fracture. This is demonstrated in the study by Hong et al., where the absence of edema and hypoechoic hematoma is identified as distinguishing features that can differentiate an old fracture line from acute trauma [26].

While the current meta-analysis provided compelling findings regarding the diagnostic efficacy of ultrasound in detecting nasal bone fractures in children, certain limitations should be acknowledged. Although the reference test across all included studies was physical examination, factors such as the operator’s expertise, patient age groups, types of fractures, criteria used for definition of fractures, transducer resolution, adherence to standardized imaging protocols, real-time visualization, interpretation of results during imaging, and the time elapsed from injury to imaging may influence the diagnostic validity of ultrasonography in detecting fractures [27]. These factors serve as potential sources of heterogeneity in the study. However, the limited number of studies on this topic constrained our ability to conduct meta-regression or subgroup analysis to assess the impact of these factors on diagnostic performance. It also remains unclear whether the results of the physical examination and medical history were blinded to the sonography operator in all the studies included in our analysis. Moreover, observer bias is a limitation attributed to the superficial nature of the nasal bone. Ultrasound operators might be influenced by visible deformities when interpreting images, potentially resulting in an overestimation of ultrasound accuracy.

Conducting additional well-designed studies with larger sample sizes and rigorous methodologies is essential. These studies should include blinding sonography operators to physical examination and medical history results, while also comparing various standardized diagnostic protocols and imaging modalities. These investigations should aim to explore the potential impact of the various factors mentioned above on the diagnostic performance of ultrasound in children with nasal bone fractures.

## Conclusion

This study highlighted the utility of ultrasonography as a diagnostic tool for pediatric nasal bone fractures, particularly due to its high accuracy and specificity. Following the exclusion of an outlier study, the analysis also revealed a notable level of sensitivity for this modality in this context. However, the suboptimal overall diagnostic performance of ultrasound, as indicated by likelihood ratios, underscores the importance of exercising caution in relying solely on ultrasound and highlights the necessity for additional diagnostic methods and further refinement in clinical practice. Researchers are encouraged to conduct additional large-scale studies to improve the generalizability of findings. Specifically, they should investigate the impact of potential factors on the diagnostic performance of ultrasound in this area, contributing more robust evidence to the field.

### Electronic supplementary material

Below is the link to the electronic supplementary material.

Supplementary Material 1

## Data Availability

The datasets analyzed during the current study are available from the corresponding author on reasonable request.

## Declarations

### Leadership role

Ali Gholamrezanezhad serves as the Director of Clinical Research at the Department of Radiology in the Keck School of Medicine of USC. Pauravi S. Vasavada holds the position of Director of Medical Student Education at Case Western Reserve University School of Medicine. Furthermore, she serves as the Co-director of the Women in Radiology Interest Group at the same institution
