# Supplementary material for: Diagnostic utility of ultrasound in pediatric nasal bone fractures: a systematic review and meta-analysis
Source: Emerg Radiol. 2024 Mar 28;31(3):417–28. doi: 10.1007/s10140-024-02225-1 (PMC11130032; doi:10.1007/s10140-024-02225-1)
Supplement: Supplementary file 1 — Supplementary Material 1 [file 10140_2024_2225_MOESM1_ESM.docx]

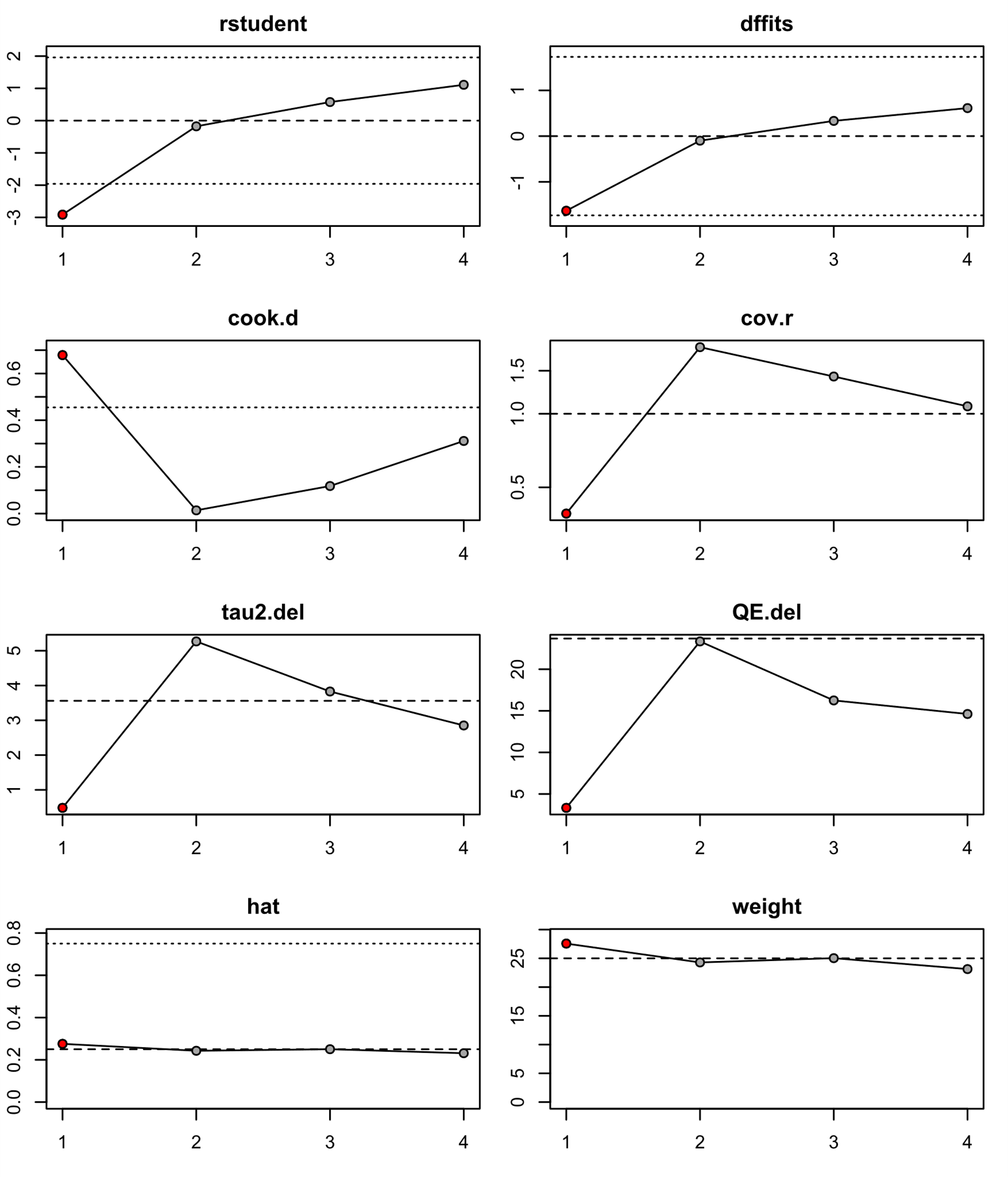


**Supplementary Fig. 1.** Influential analysis plot for the random-effects univariate diagnostic odds ratio meta-analysis.


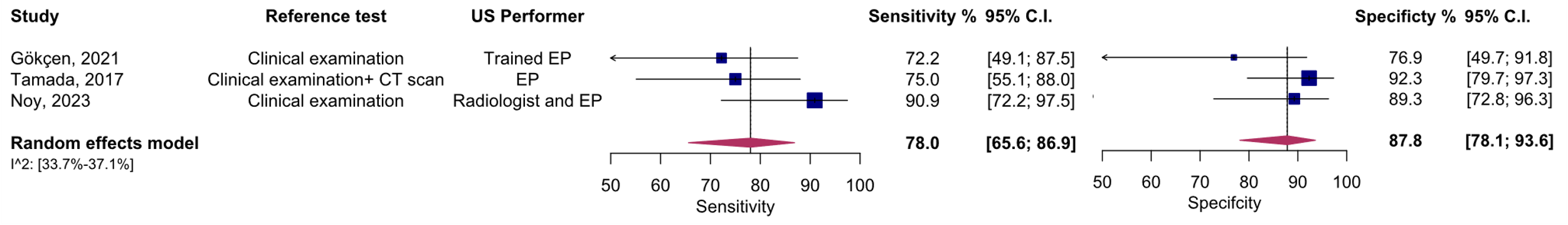


**Supplementary Fig. 2.** Forest plot and summary statistics of diagnostic test accuracy (DTA) meta-analysis, after excluding one outlier study. CI. Confidence interval. EP. Emergency practitioner.


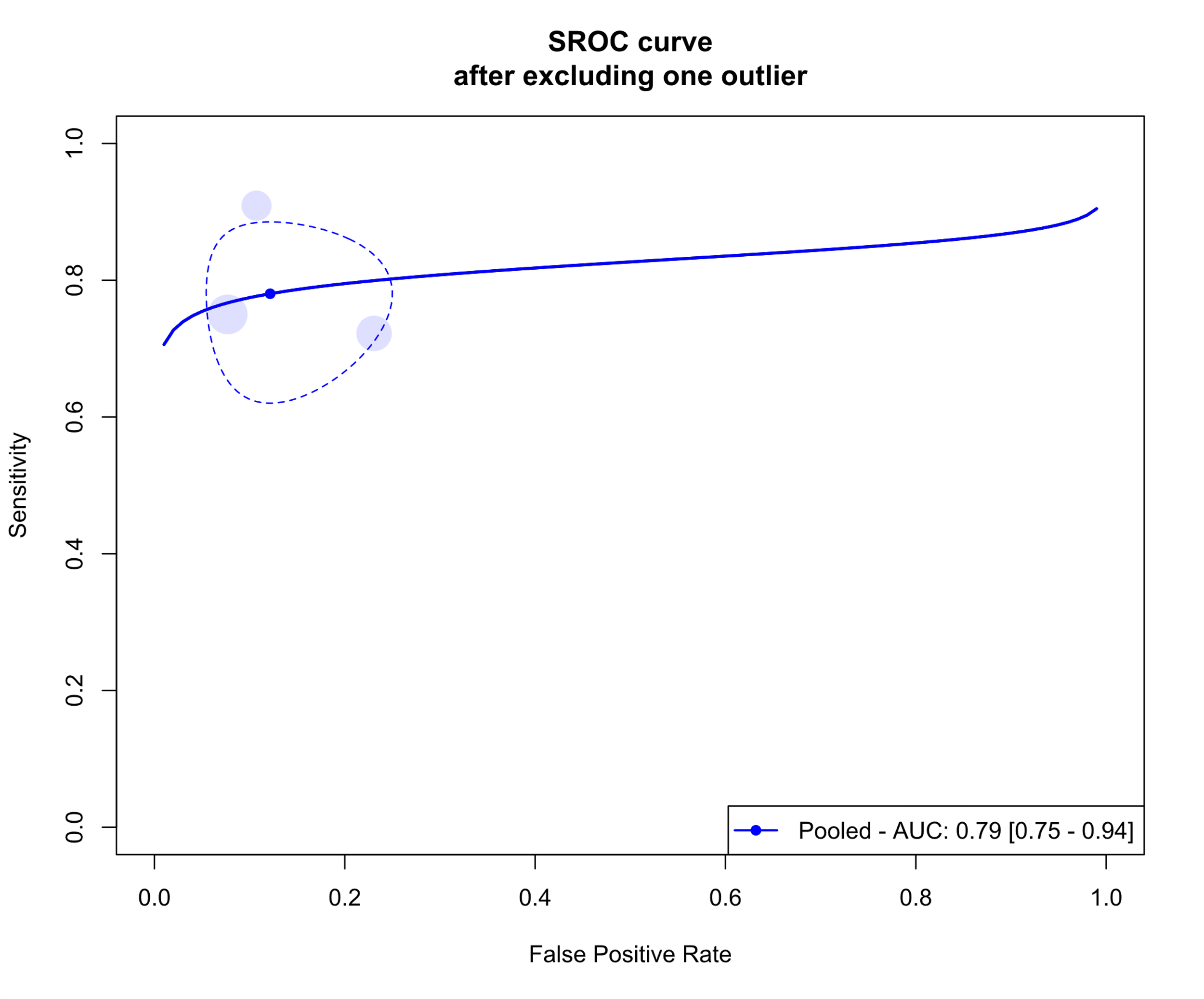


**Supplementary Fig. 3.** Summary receiver operating characteristic curve (SROC) of diagnostic test accuracy (DTA) meta-analysis, after excluding one outlier study. AUC. Area under the curve. SROC. Summary receiver operating characteristic.


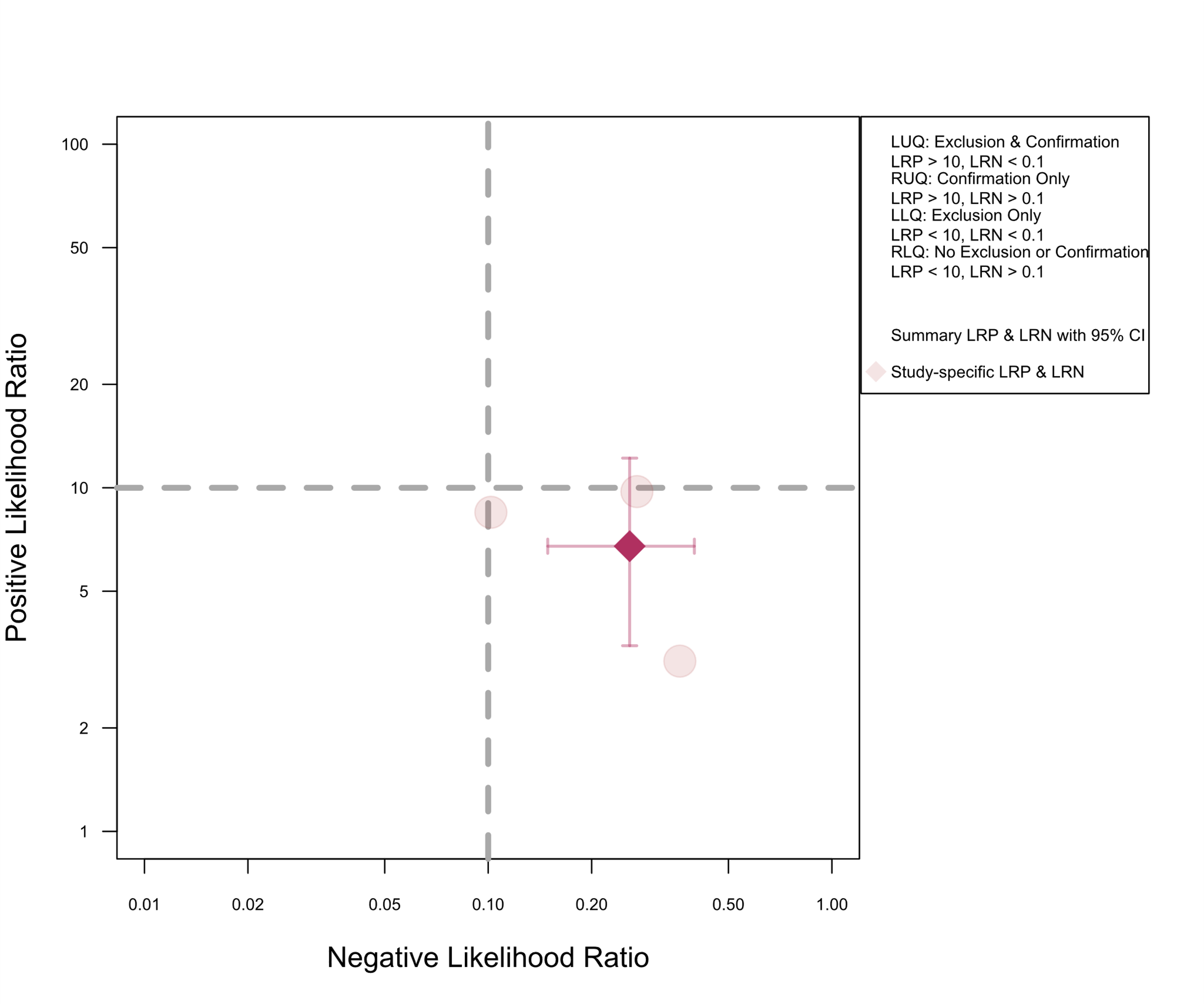


**Supplementary Fig. 4.** Likelihood ratio scattergram after excluding one outlier study, indicating low to moderate test performance suboptimal for both exclusion and confirmation purposes. LLQ. Left lower quadrant. LRN. Likelihood ratio, negative. LRP. Likelihood ratio, positive. LUQ. Left upper quadrant. RLQ. Right lower quadrant. RUQ. Right upper quadrant.


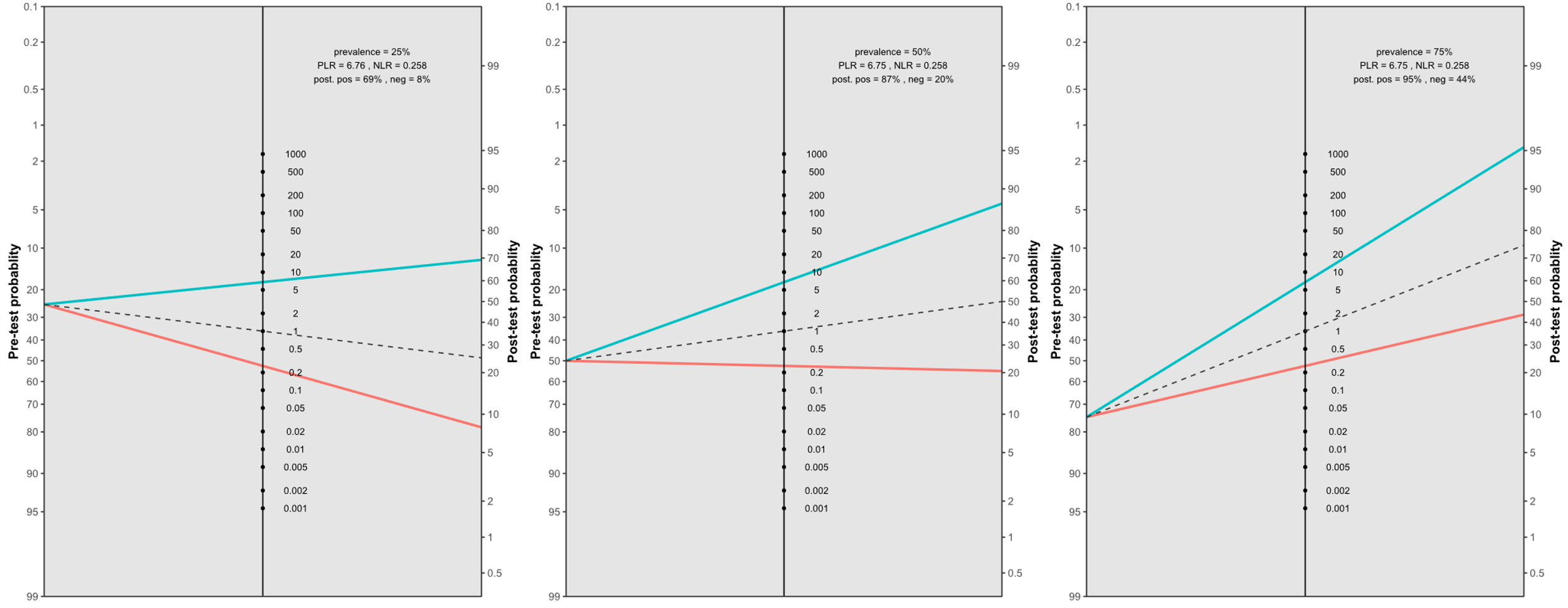


**Supplementary Fig. 5.** Fagan plot analysis utilizing summary positive and negative likelihood ratio results from the meta-analysis after excluding one outlier study, considering hypothetical pre-test probabilities of 25%, 50%, and 75%. PLR. Positive likelihood ratio. NLR. Negative likelihood ratio. Neg. Negative. Pos. Positive
